# BCL-2 expression aids in the immunohistochemical prediction of the Oncotype DX breast cancer recurrence score

**DOI:** 10.1186/s12907-018-0082-3

**Published:** 2018-12-18

**Authors:** Mark D. Zarella, Rebecca C. Heintzelman, Nikolay K. Popnikolov, Fernando U. Garcia

**Affiliations:** 1 Department of Pathology & Laboratory Medicine, Drexel University, 245 N 15th St, Philadelphia, PA 19102 USA; 2 Cancer Treatment Centers of America, Eastern Regional Medical Center, Department of Pathology & Laboratory Medicine, 1331 E. Wyoming Ave, Philadelphia, PA 19124 USA

**Keywords:** Digital pathology, Prognostic markers, Computer-assisted diagnosis, Staining

## Abstract

**Background:**

The development of molecular techniques to estimate the risk of breast cancer recurrence has been a significant addition to the suite of tools available to pathologists and breast oncologists. It has previously been shown that immunohistochemistry can provide a surrogate measure of tumor recurrence risk, effectively providing a less expensive and more rapid estimate of risk without the need for send-out. However, concordance between gene expression-based and immunohistochemistry-based approaches has been modest, making it difficult to determine when one approach can serve as an adequate substitute for the other. We investigated whether immunohistochemistry-based methods can be augmented to provide a useful therapeutic indicator of risk.

**Methods:**

We studied whether the Oncotype DX breast cancer recurrence score can be predicted from routinely acquired immunohistochemistry of breast tumor histology. We examined the effects of two modifications to conventional scoring measures based on ER, PR, Ki-67, and Her2 expression. First, we tested a mathematical transformation that produces a more diagnostic-relevant representation of the staining attributes of these markers. Second, we considered the expression of BCL-2, a complex involved in regulating apoptosis, as an additional prognostic marker.

**Results:**

We found that the mathematical transformation improved concordance rates over the conventional scoring model. By establishing a measure of prediction certainty, we discovered that the difference in concordance between methods was even greater among the most certain cases in the sample, demonstrating the utility of an accompanying measure of prediction certainty. Including BCL-2 expression in the scoring model increased the number of breast cancer cases in the cohort that were considered high certainty, effectively expanding the applicability of this technique to a greater proportion of patients.

**Conclusions:**

Our results demonstrate an improvement in concordance between immunohistochemistry-based and gene expression-based methods to predict breast cancer recurrence risk following two simple modifications to the conventional scoring model.

## Background

A number of prognostic factors have been described with a significant relationship to likelihood of breast cancer recurrence, and commonly include patient age [1, 2], tumor size [3], lymph node status [4], and histologic grade [5], but also include biomarkers as assessed by immunohistochemistry (IHC) including Her2 status [6], patterns of hormone receptor expression (ER and PR) [7, 8], and expression of proliferative marker Ki-67 [9, 10]. Gene expression analysis has also been used to successfully predict recurrence likelihood [11], with one such test (Oncotype DX, Genomic Health, Redwood City, CA) having been added to NCCN guidelines for treatment [12]. This test provides a recurrence score (RS) which estimates the likelihood of recurrence according to a 21-gene assay. Importantly, RS has also been shown to successfully predict chemotherapy benefit [13], and has extensively been shown to alter treatment recommendations when considered alongside other diagnostic factors [14–20].

Despite the clear clinical utility of this approach, it remains costly and inaccessible to many patients, leading several groups to evaluate alternative methods as a surrogate for the Oncotype DX test. Turner, et al. suggested that limiting the use of the test based on routine histologic examination could reduce send out by up to 23%, resulting in significant cost savings [21]. More recently, Gage, et al. argued that a 44% reduction in send out could be achieved based on traditional variables used in sign out [22]. In addition to economic considerations, another focus has been to provide accurate predictions from immediately available data. Flanagan, et al. described one of the most compelling methods, using histologic factors combined with routinely acquired protein expression data from IHC to predict RS [23]. This approach provided an estimate of recurrence likelihood using data immediately available following routine processing of the tissue. In a subsequent study, this group offered three modifications to the original formulation with similar predictive success [24]; one such modification, the *Magee Score #3*, generated a prediction of RS strictly from ER, PR, Ki-67, and Her2 status. An advantage to this modification is that it is entirely quantitative and reproducible, and does not depend on pathologist grading or interpretation which has been shown to exhibit considerable inter-observer variability [25]. Furthermore, with the advent of whole-slide imaging and its association with modern informatics approaches, a score based entirely on image analysis of IHC has the potential to be generated in a completely automated fashion.

Although the *Magee Score #3* is an attractive alternative to the Oncotype DX test, it achieved only 54.4% overall concordance in their study. Improved concordance between this IHC-based approach and RS is needed to inspire confidence in the performance of this technique. We explored whether two modifications to this approach could be used to improve concordance and produce a viable IHC-based algorithm with clinical potential. First, we examined the impact on concordance by applying a diagnostically-relevant data transformation. Second, we explored whether the expression of BCL-2 as part of a complete breast panel can improve the estimation of RS. BCL-2 expression has previously been shown to be associated with a decreased risk of recurrence [26, 27] and a higher relapse-free survival rate [28–32]. We hypothesized that the prognostic information provided by BCL-2 accompanied by an optimized mathematical treatment of these variables can be harnessed to improve RS estimation.

## Methods

### Case selection

One hundred and fifty-eight breast cancer cases between 2010 and 2017 were identified retrospectively from the Drexel University College of Medicine breast cancer databank that met the following criteria:

1. a diagnosis of primary invasive breast carcinoma was rendered on the original pathology report;
2. Oncotype DX recurrence scores were available in the original pathology report;
3. a breast panel that included expression of ER, PR, Ki-67, Her2, and BCL-2, had been performed on the same block using IHC;
4. slides were scanned using whole-slide imaging and staining was assessed quantitatively using computational image analysis;
5. ER percent positive staining was at least 1%;
6. Her2 was considered negative or equivocal by image analysis and confirmed manually.

Case data were obtained by an honest broker and delivered to the investigators in a deidentified fashion. This study was considered exempt by the Drexel University College of Medicine Institutional Review Board under Category 4.

### Histologic processing

All breast specimens had ischemic and fixation times within CAP/ASCO guidelines. Biopsy cases were processed in a standard fashion after fixation in neutral buffered formalin. Lumpectomy specimens were entirely submitted after fixation in neutral buffered formalin and inking all surfaces to maintain orientation. A specimen map was used as a worksheet to document lesional tissue and distance to margins. After review of all carcinoma slides, the most representative slide was used to perform the invasive breast panel consisting of ER, PR, Ki-67, Her2, and BCL-2. Immunohistochemical stains for ER (SP1, RM, Ventana, Benchmark Ultra), PR (1E2, RM, Ventana, Benchmark Ultra), Ki-67 (30–9, RM, Ventana, Benchmark Ultra), Her2 (4B5, RM, Ventana, Benchmark Ultra), and BCL-2 (124, MM, Ventana, Benchmark Ultra) antibodies were performed on formalin-fixed paraffin-embedded sections. The antibody conditions for ER, PR, Ki-67, Her2, and BCL-2 were as follows: 8.1 pH antigen retrieval using CC1 reagent for 36–64 min, followed by primary antibody incubation for 16–44 min, and then staining with the Ultra Ultraview Universal DAB Detection Kit.

### Image analysis

High resolution whole-slide images were acquired at either 20x or 40x magnification using the Aperio Scanscope XT (Leica Microsystems, Wetzlar, Germany). Eight regions of interest were manually selected by a pathologist for scoring, and the average score was derived using one of three semi-automated algorithms provided by the Aperio software. The nuclear staining algorithm was applied to ER, PR, and Ki-67 slides and was used to compute the percentage of positively stained cells, as well as an H-score indicating staining intensity, consistent with CAP/ASCO guidelines [33]. The membrane algorithm was applied to Her2 slides and generated a Her2 score consistent with CAP/ASCO guidelines [34]. The cytoplasmic algorithm was applied to BCL-2 slides and produced an H-score based on cytoplasmic staining intensity. Importantly, these scores were obtained at the time of diagnosis and were not influenced by the purposes of this study. Original Her2 scores that were reported using previous CAP/ASCO guidelines [35] were recomputed to meet current standards, but this did not require the image analysis portion to be modified.

### Regression model

We performed linear regression to derive a set of coefficients (and a constant term) that, in combination with selected IHC data, could be used to predict RS. To compute concordance, we embedded this model within a 10-fold cross-validation framework to ensure that the test sample was not used to generate the coefficients. To further ensure that the model was not susceptible to overfitting, we repeated the cross-validation 10,000 times, randomly selecting training and test data groups (folds) at each iteration. The results that we report are accompanied by a standard deviation which represents the iteration-to-iteration variability of the value under test. Generally, we observed only a small difference in the model’s coefficients between iterations.

### Logistic transformation

Using Eq. 1, we applied a logistic transformation to the percent positive nuclear staining metric, *x*, to generate a diagnostically-relevant score, *y*, between zero and one:

1 $$ y=\frac{2}{1+{e}^{- kx}}-1 $$

The value, *k*, was selected so that *y* is approximately half its maximum at the diagnostic threshold, *T*_*x*_ (Eq. 2).

2 $$ k=\frac{\ln 3}{T_x} $$

This relationship holds true only for small values of *T*_*x*_ (i.e. when *e*^*-k*^ approaches zero). For example, when *T*_*x*_ is 0.14, *y* is equal to 0.500 when *x* is 0.14 and 0.999 when *x* is 1. We selected values of *T*_*x*_ based on previous reports of diagnostic criteria that were successful in stratifying patients using ER [36], PR [37], and Ki-67 [9, 10, 38] (10, 10, and 14%, respectively).

### Analysis of concordance

We measured concordance between the IHC-based score we generated and RS by comparing the categorical interpretations of both scores. We judged the certainty, *η*, of the categorical prediction by measuring the difference between the score and its closest categorical threshold (Eq. 3).

3 $$ \eta =\mathit{\min}\left(\left|y-18\right|,\left|y-31\right|\right) $$

We used a threshold of 18 to distinguish the low from intermediate group and a threshold of 31 to distinguish the intermediate from high group, consistent with thresholds established for the Oncotype DX test [11].

### Relationship between H-score and percent positive cells

We used H-score to describe the staining attributes of ER and PR in a subset of analyses. We observed that H-score, in contrast to the logistic score described above, maintained a linear relationship with the percent positive metric. To demonstrate the relationship between H-score and the percent positive metric, we performed simulations by randomly assigning a staining intensity of 0, 1+, 2+, or 3+ to a set of 10^4^ model cells. We measured the H-score and computed the corresponding percent positive staining for the model cells, repeating this process 10^5^ times.

## Results

### The conventional scoring model is highly reproducible

We performed a retrospective analysis of 158 cases (103 resections, 55 core needle biopsies) of invasive breast carcinoma. As shown in Table 1, relative proportions of histologic grade in this sample were consistent with previous reports [39, 40]. Furthermore, the relative proportions of histologic grade were roughly equivalent across both biopsies and resections, confirming that a bias did not exist as a function of sampling procedure. Cases with histologic grade of 3 also had higher Oncotype DX recurrence scores (RS) than cases with a grade of 2 (*p* < < 0.01, Mann-Whitney U test), as demonstrated previously [41–45]. The proportions of PR-positive and Ki-67-positive cases present in this ER-positive and Her2-negative/equivocal sample were also found to be within an expected range [46, 47] (Table 2).

**Table 1:** Case details by histologic grade

		Histologic grade		
	Total	I	II	III
Number of cases	158	34	86	38
Resection	103	20	58	25
Biopsy	55	14	28	13
Median patient age	57 ± 8	57.5 ± 6	56.5 ± 7	56.5 ± 9
Tumor size (cm)	1.6 ± 0.6	1.5 ± 0.7	1.6 ± 0.7	1.5 ± 0.4
Oncotype DX RS	15 ± 4.5	14 ± 3	14 ± 4	23.5 ± 6.5

The number of cases in our cohort are divided by histologic grade and presented according to relevant clinicopathological variables, including RS. Ranges represent quartiles

**Table 2:** Immunohistochemistry attributes of the data set

	Negative or Not overexpressed	Low positive or Equivocal	Positive or Overexpressed
ER	0 (0.0%)	2 (1.3%)	156 (98.7%)
PR	14 (8.9%)	12 (7.6%)	132 (83.5%)
Ki-67	34 (21.5%)	46 (29.1%)	78 (49.4%)
Her2	134 (84.8%)	24 (15.2%)	0 (0%)

The relative proportions of ER, PR, Ki-67, and Her2 categories are shown as the number (and corresponding percentage) of cases

We developed an equation using linear regression to predict breast cancer recurrence by estimating RS from ER, PR, Ki-67, and Her2 expression determined using IHC. We categorized IHC scores using a threshold of 18 to distinguish between “low” and “intermediate” risk of recurrence and 31 to distinguish between “intermediate” and “high” risk of recurrence, the same thresholds used for RS categorization [13]. We measured the concordance between RS and the categorical predictions of IHC using the H-scores of ER and PR, the percent positive staining for Ki-67, and Her2 category (overexpressed, not overexpressed, equivocal), following previous methods [23, 24, 48, 49]. We found that IHC and RS shared the same category in 63.4% of cases. In comparison, we used the coefficients from the *Magee Score #3* [24] which was based on the same variables, and found similar overall performance to our eq. (59.5%), a rate that exceeded the performance reported by the authors when applied to their cohort of 248 cases (54.4%). We also compared the coefficients of both equations and found that they were similar (Table 3, first and second columns) except that Her2 status was more strongly weighted in the equation derived from our cohort, likely due to the difference in the Her2 scoring standard applied. Nevertheless, these results demonstrate a high degree of reproducibility of the conventional scoring model on the data set presented here and confirm its ability to predict RS.

**Table 3:** Contribution to IHC score

	Conventional model		Revised model	
	Magee Score #3 coefficients	Linear regression coefficients	Linear regression coefficients	Standardized weight
ER	−0.022	−0.025	−26.75	2.28
PR	−0.029	−0.040	−10.83	3.59
Ki-67	0.186	0.196	11.64	3.15
Her2	1.47^a^	7.97^a^	9.14	2.06
BCL-2	–	–	−7.39	2.17

^a^ Her2 score was defined differently in our calculation of Her2 score, reflecting the change in Her2 recommendations from Hammond, et al., 2013

The relative contributions of each marker to the IHC-based scores are presented using metrics that examine the weighting of each quantity in the equations. The conventional model refers to the model based on the technique described in Klein, et al. using ER and PR H-score, Ki-67% positive cells, and Her2 score. The conventional model uses a variable weight based on the categorical interpretation of Her2; for clarity, only the coefficient that is applied to a Her2 score of 2 is shown. The equation’s coefficients derived from the Klein data set are shown in the first column and the coefficients that we derived from our cohort are shown in the second column. Notably, BCL-2 is excluded from this analysis because it was not a variable included in the authors’ formulation of the equation. The coefficients from the revised model, which includes BCL-2 as a prognostic variable, ER/PR percent positive cells as a replacement for H-score, and follows the logistic transformation applied to ER, PR, and Ki-67, and a linear treatment of the Her2 score, are shown in the third column. The standardized weights of the markers, defined as the product of the coefficients and the standard deviations of the values in our cohort, are shown in the fourth column

### Transformation of staining attributes improve concordance

CAP/ASCO guidelines specify that ER and PR expression as determined by IHC should be reported according to the percentage of positively stained cells to guide diagnostic interpretation and treatment decision making [33]. Likewise, Her2 expression is reported according to a score which measures the relative proportions of 3+, 2+, 1+, and unstained cells based on membrane staining intensity [34]. Treatment decision making critically relies on the categorical interpretation of these quantities [50–55], but it is difficult to reconcile the clinical utility of categorical data with the linear treatment of a continuous variable. For instance, a tumor is considered to exhibit ER immunoreactivity if the percentage of positively stained cells exceeds 1% [33]. This implies that the binary categorization of this quantity into “positive” and “negative”, which has a profound effect on its diagnostic and therapeutic interpretation, largely ignores differences in expression levels over the vast majority of its range. Therefore, using a continuous scalar quantity such as percent positive cells or H-score to characterize ER expression is not consistent with the diagnostic interpretation of ER.

**Fig. 1:**
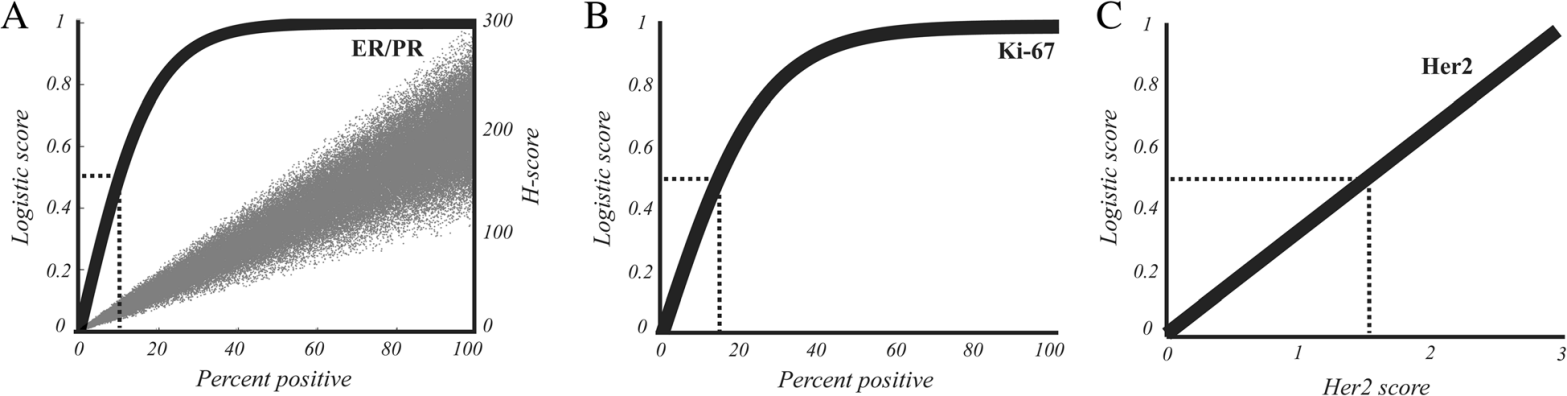
Transformation of staining attributes. **a** The ER and PR percent positive cells metric was transformed to a diagnostic score using logistic transformation. As indicated by the dotted lines, the curve is half maximum at the diagnostic cut-off of 10%, producing a score of 0.5. Gray points indicate the relationship between H-score and percent positive cells using simulations. **b** The logistic transformation is applied to the Ki-67% positive cells metric using a diagnostic cut-off of 14% to produce a diagnostic score. **c** Her2 score is transformed to a diagnostic score by dividing its value by 3, maintaining a linear relationship

We developed a scoring model in which we applied a logistic transformation to the percent positive metric to produce a value that is consistent with the diagnostic interpretation of ER, PR, and Ki-67. This transformation produced a diagnostically-relevant score whose relationship to percent positivity is shown in Fig. 1, in contrast with the H-score (Fig. 1a, gray points) which maintained a linear relationship with percent positivity. Likewise, we removed the tripartite categorization of Her2 score to better reflect the prognostic interpretation of Her2 status, given that Her2 scores of 1 have been shown to predict a significantly higher incidence of recurrence than scores of 0 [56] despite both being assigned to the same “not overexpressed” category that is largely used to determine eligibility for Her2-specific treatment. After applying these transformations, we again computed predictive scores using cross-validation. We found that 64.6% of cases were assigned by IHC to the same category as the corresponding RS, a modest improvement over the 63.6% concordance rate achieved by not using logistic transformation.

### BCL-2 improves classification performance

**Fig. 2:**
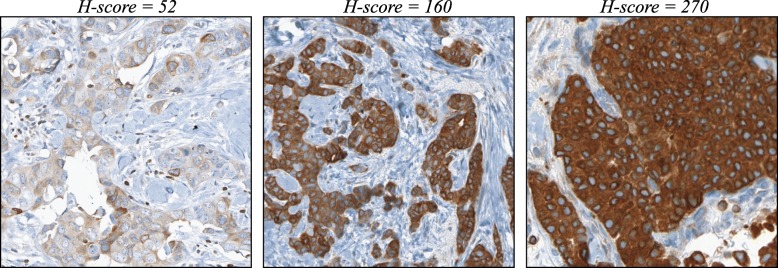
Representative examples of BCL-2 staining.We selected representative images from three cases that exhibited low (left panel), intermediate (center panel), and high (right panel) intensity staining for BCL-2. The corresponding H-scores are shown

**Fig. 3:**
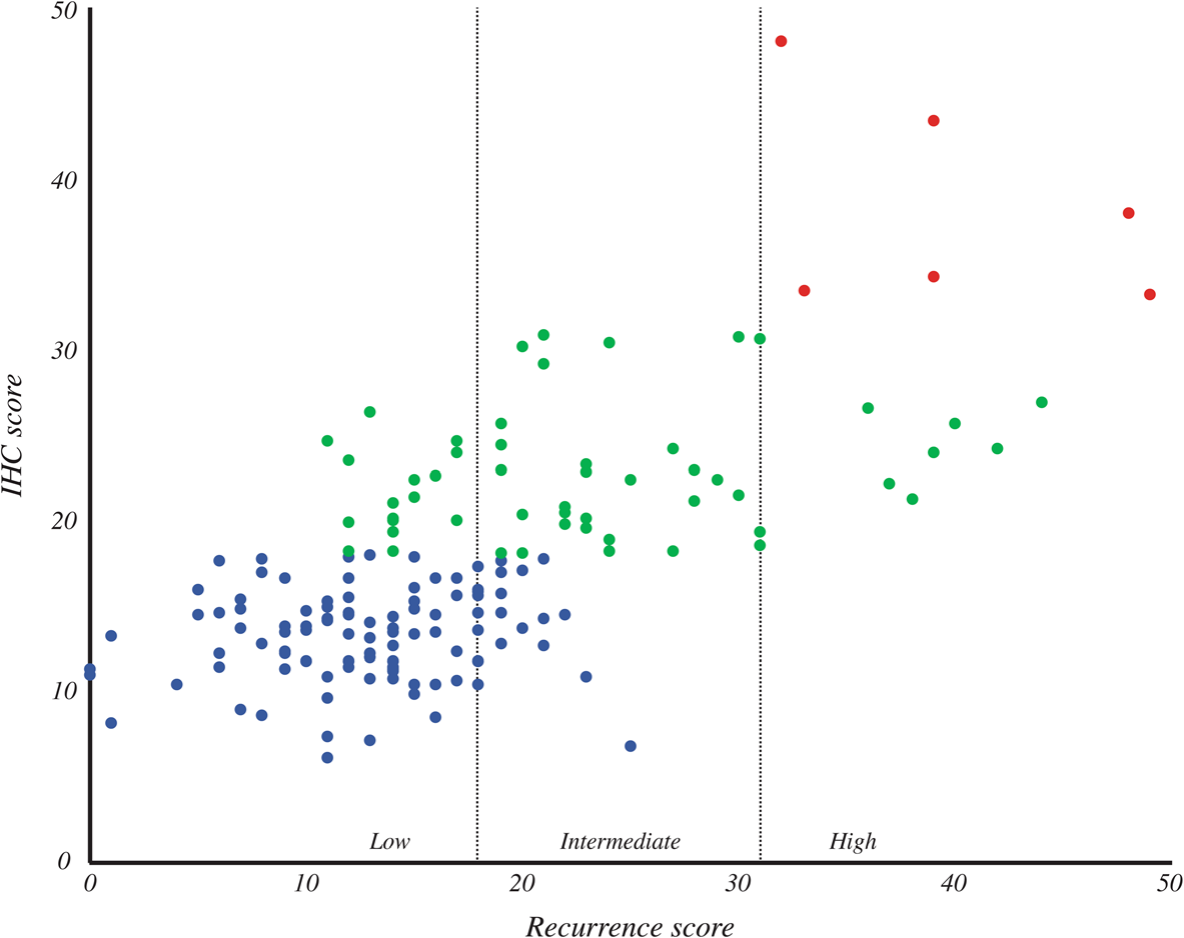
Estimation of RS from IHC. The RS for each sample is represented on the x-axis. The IHC scores generated by linear regression, following data transformation and the addition of BCL-2, are represented on the y-axis. Dotted lines represent the categorical boundaries that distinguish low, intermediate, and high risks of recurrence. The category to which each sample is assigned based on IHC score is indicated by color; blue = low, green = intermediate, red = high

**Fig. 4:**
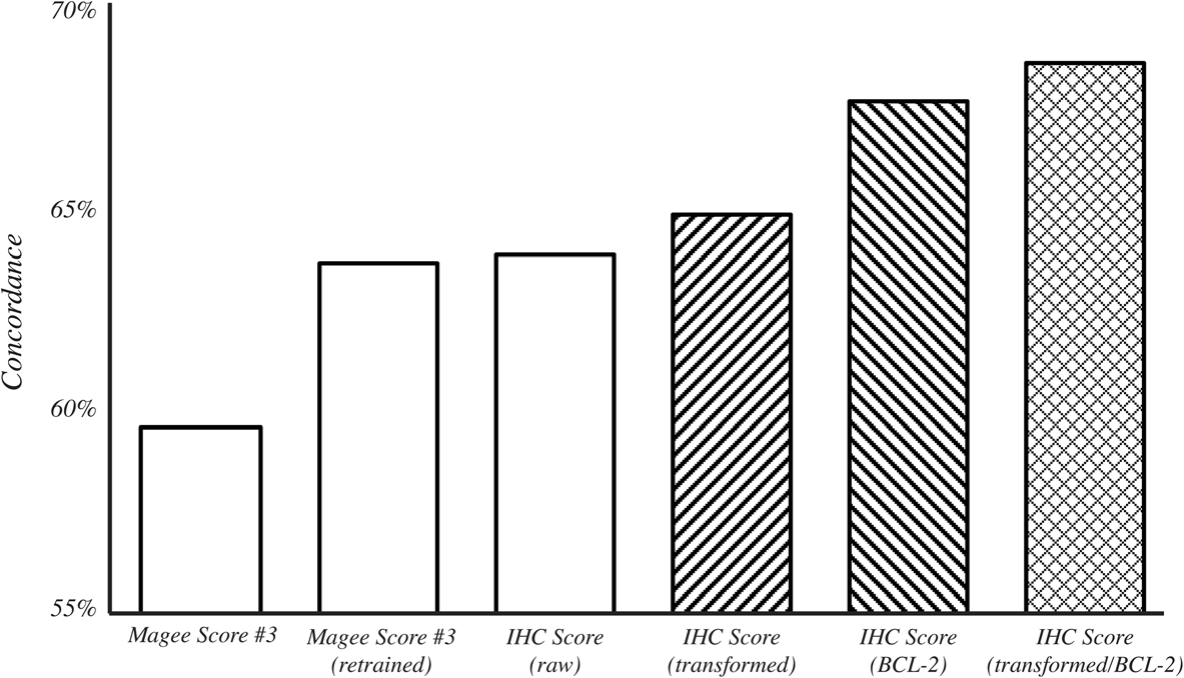
Concordance of IHC-based methods with RS. Concordance rates were computed on 158 cases using cross-validation for each of the following methods tested (starting with the left-most bar): *Magee Score #3* with the coefficients and variables described in Klein, et al. [24]; *Magee Score #3* with coefficients recomputed based on our cohort; IHC Score using ER, PR, and Ki-67% positive staining, and Her2 score; IHC Score using the logistic transformation applied to ER, PR, and Ki-67; IHC Score after inclusion of BCL-2 H-score; IHC Score using both the logistic transformation and the inclusion of BCL-2 H-score

Low BCL-2 expression in breast cancer has been associated with increased likelihood of recurrence [26, 27] and generally carries a less favorable prognosis [28–32]. We augmented the IHC-based methods by adding BCL-2 expression as an additional prognostic variable. Since there is not an established threshold for stratifying recurrence risk for BCL-2, we elected to use the H-score to quantify BCL-2 staining (Fig. 2) instead of applying the logistic transform with an arbitrary threshold. In Fig. 3, the distribution of IHC-based scores using ER, PR, Ki-67, Her2, and BCL-2 is shown in relation to the corresponding RS. We again categorized IHC-based scores into “low”, “intermediate”, and “high” risk groups. Using these criteria, blue points represent scores that were classified by IHC as low risk; likewise, green points and red points represent scores in the intermediate and high risk categories, respectively. We found that the addition of BCL-2 further improved the concordance rate to 68.2% (Fig. 4, rightmost bar).

**Fig. 5:**
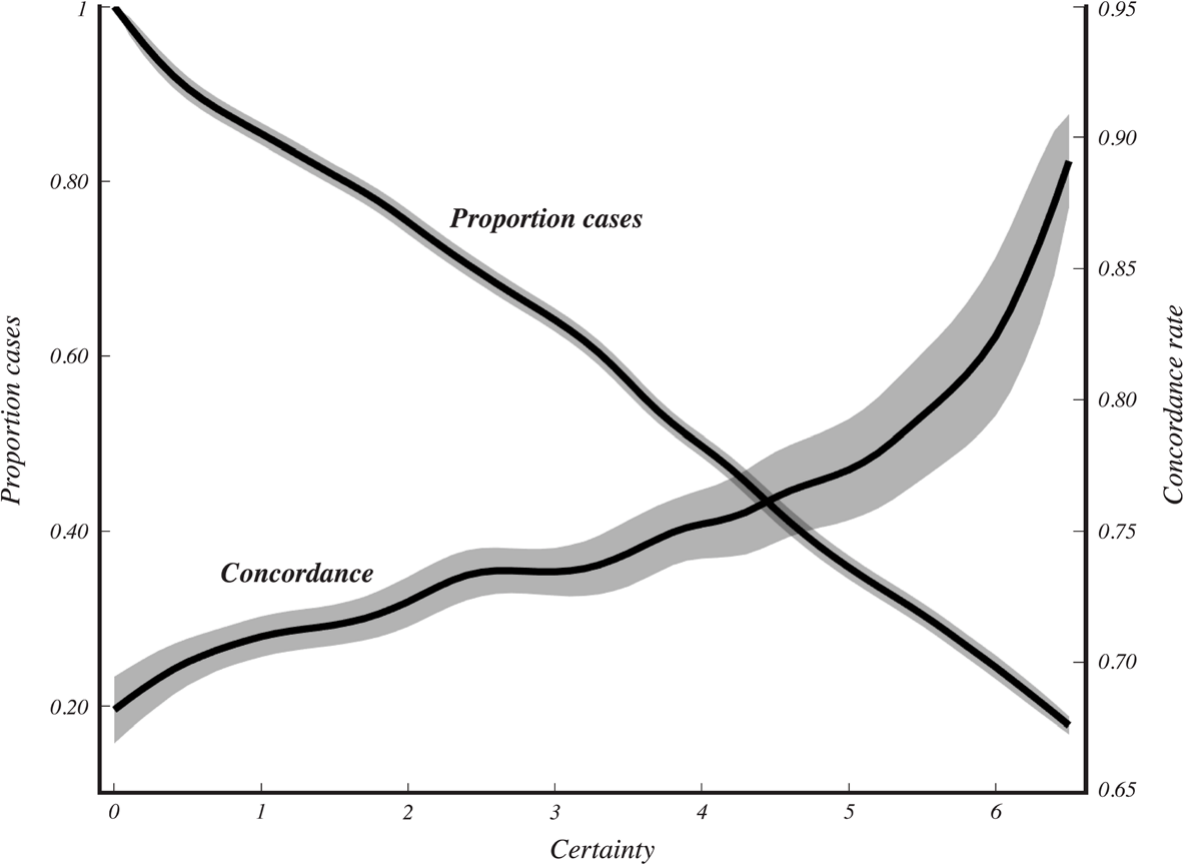
Cumulative concordance rate scales with certainty. Concordance rates were computed based only on the samples with certainty values greater than the value indicated on the x-axis. The proportion of cases used to compute the concordance value is shown on the y-axis to the left, where 1 indicates that all 158 samples were used. Standard deviation of the rates based on 10,000 iterations are represented by the shaded regions

To further demonstrate the impact of these modifications on concordance rate, we sorted the IHC score as a function of prediction certainty. We hypothesized that scores close to 18 or 31 have a higher likelihood of misclassification due to their proximity to the categorical boundaries. We developed a measure of certainty based on the difference between the score and its closest threshold. We found that the most certain predictions were indeed more likely to be concordant with RS. However, cases with IHC scores close to 18 were always either low or intermediate according to RS and cases with IHC scores close to 31 were always either intermediate or high. We did not observe any cases that were classified into the low category by IHC and the high category by RS, or vice versa. As shown in Fig. 5, the likelihood of concordant classification increased when only the most certain cases were considered, achieving a concordance of nearly 90% for the most certain 20% of the cohort.

### Individual contribution of markers to the prediction of RS

We compared the independent contributions of each marker to the concordance rate of the algorithm. When markers were individually used to predict RS, we found that they were poor predictors alone. When we evaluated the performance of the algorithm when trained with only two markers at a time, we found that PR and BCL-2 together produced the highest number of high-certainty scores (> 3), achieving an overall concordance rate of 63.7%. We found that the most successful trio of markers was PR, BCL-2, and Ki-67, expanding the number of high-certainty predictions and achieving an overall concordance rate of 65.3%.

We developed an alternative measure of the individual contributions of each marker by examining the equation’s coefficients. We found that the standardized weights of the coefficients were similar to the rank order observed originally, except that Ki-67 had a much higher standardized weight than BCL-2 (Table 3, fourth column). The disparity between the results of the two methods indicates that BCL-2 and PR likely share a complementary role that is not revealed when all the markers are present.

### IHC score to predict chemotherapy benefit

**Fig. 6:**
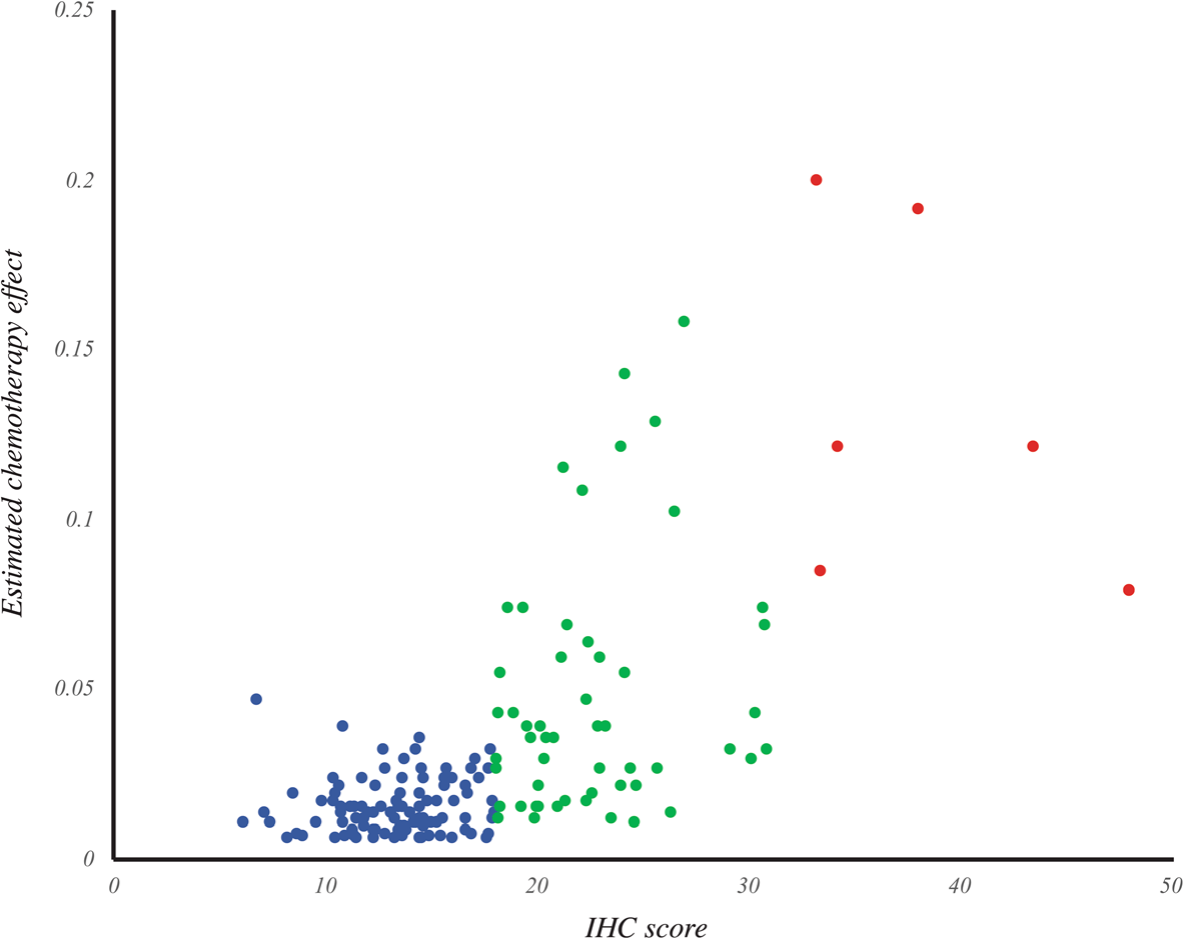
Prediction of chemotherapy effect from IHC. Chemotherapy effect was calculated from RS and plotted against IHC score for each case. Chemotherapy effect was estimated from the model presented by Paik, et al. [13] by subtracting the 10-year likelihood of recurrence at a given RS for patients treated with Tamoxifen + chemotherapy from those treated with Tamoxifen alone. The category to which each sample is assigned based on IHC score is indicated by color; blue = low, green = intermediate, red = high

These results, taken together with those that have examined chemotherapy benefit as a function of RS [13, 16, 20, 57–59], suggest that patients with a sufficiently low IHC score are unlikely to benefit from chemotherapy. Recent evidence from the TAILORx trial suggests that a subset of patients with RS scores in the intermediate range are also unlikely to benefit from chemotherapy [60]. Figure 3 shows that of the 98 cases with IHC scores in the “low” group, only 21 (21.4%) equaled or exceeded an RS of 18 that would put them into the intermediate category. Furthermore, these 21 discordant cases had a mean RS of 19.6, suggesting that they predominantly resided on the low end of the intermediate range. Only 3 of the 98 cases classified by our technique as belonging to the “low” group had an RS greater than 21. Therefore, very few of the patients misclassified by the IHC score are likely to benefit from chemotherapy. We replotted the data in Fig. 3 and expressed the results in terms of estimated chemotherapy benefit (Fig. 6). The results support our contention that patients with tumors that were classified into the “low” category using IHC were unlikely to exhibit a chemotherapy benefit. Based on these results, we suggest that patients with an IHC score less than 18 do not benefit from additional molecular testing to confirm that they are unlikely to benefit from chemotherapy. In our cohort, this cutoff would reduce send-out by 62%.

## Discussion

The results that we describe demonstrate a quantitative approach to estimating the likelihood of breast cancer recurrence from routinely acquired protein expression data. This technique offers the ability to immediately stratify patients following IHC processing of excised tissue. The IHC score is based strictly on the results of image analysis of tissue and does not require other information that may not be available at the time of biopsy, for example. It also does not necessarily require whole-slide imaging; it can be used with any semi-quantitative method for estimating staining and therefore is accessible to underprivileged areas, offering a key advantage over some molecular approaches. However, when combined with whole-slide imaging, it may offer the potential to be robust in the presence of tumor heterogeneity by enabling the analysis to be performed with spatial precision. This not only provides a check that can confirm the reliability of the molecular interpretation of the result, but can also help guide microdissection to improve sampling for molecular send out.

The two main innovations over existing IHC-based approaches are the addition of BCL-2 to the panel and a logistic transformation pre-processing step to transform conventionally reported values into a more diagnostically-relevant score. Together, these additions improved the concordance rate from 59.5% using the *Magee Score #3* to 68.2%. Importantly, the results demonstrate that the logistic transformation step eliminates the need to use H-score to quantify ER and PR expression, which may not always be accessible. Furthermore, the IHC score can also be accompanied by a measure of certainty that describes the likelihood of the score belonging to a particular category. After converting a given score’s certainty value to a likelihood measure (Fig. 4), a sample reporting of the results may take the form:

*IHC score: 11.6*

*A score of 11.6 indicates an 89% chance of belonging to the low recurrence likelihood group and an 11% chance of belonging to the intermediate recurrence likelihood group.*

Refining the certainty and prediction accuracy measures on which these interpretations are based would be aided by testing this technique on additional data sets.

### BCL-2 as a predictive marker

Our analysis of the individual contributions of each marker to the predictive success of the IHC score suggests that all the markers we used in this study provide unique information that aids in the prediction of RS. However, the results also indicate that only two markers (PR and BCL-2) are needed to provide approximately the same performance as conventional IHC measures for recurrence likelihood. This result illustrates the complementary contributions of PR and BCL-2 in predicting recurrence, and perhaps de-emphasizes the importance of ER, Ki-67, and Her2 in this role. Although ER, Ki-67, and Her2 are routinely analyzed in invasive breast cancer cases, the results also suggest that quantitative image analysis can likely be sequestered to just PR and BCL-2 slides in underprivileged areas for the purpose of predicting breast cancer recurrence.

## Conclusions

We suggest that conventional methods for estimation of the Oncotype DX breast recurrence score can be augmented by the addition of BCL-2, by the mathematical transformation of ER, PR, and Ki-67, or by the combination of both approaches. The results indicate that this modification improves concordance between IHC- and gene-expression-based methods, and can be extended to predict chemotherapy effect.

## Abbreviations

IHC: Immunohistochemistry
RS: Recurrence score
